# Forecasting High-Risk Behavioral and Medical Events in Children with Autism through Analysis of Digital Behavioral Records

**DOI:** 10.1101/2024.05.06.24306938

**Published:** 2024-05-06

**Authors:** Yashar Kiarashi, Johanna Lantz, Matthew A Reyna, Conor Anderson, Ali Bahrami Rad, Jenny Foster, Tania Villavicencio, Theresa Hamlin, Gari D Clifford

**Affiliations:** 1 Department of Biomedical Informatics, Emory University, Atlanta, GA; 2 The Center for Discovery (TCFD), Harris, NY; 3 Department of Biomedical Engineering, Georgia Institute of Technology, Atlanta, GA

## Abstract

Individuals with Autism Spectrum Disorder may display interfering behaviors that limit their inclusion in educational and community settings, negatively impacting their quality of life. These behaviors may also signal potential medical conditions or indicate upcoming high-risk behaviors. This study explores behavior patterns that precede high-risk, challenging behaviors or seizures the following day. We analyzed an existing dataset of behavior and seizure data from 331 children with profound ASD over nine years. We developed a deep learning-based algorithm designed to predict the likelihood of aggression, elopement, and self-injurious behavior (SIB) as three high-risk behavioral events, as well as seizure episodes as a high-risk medical event occurring the next day. The proposed model attained accuracies of 78.4%, 80.68%, 85.43%, and 69.95% for predicting the next-day occurrence of aggression, SIB, elopement, and seizure episodes, respectively. The results were proven significant for more than 95% of the population for all high-risk event predictions using permutation-based statistical tests. Our findings emphasize the potential of leveraging historical behavior data for the early detection of high-risk behavioral and medical events, paving the way for behavioral interventions and improved support in both social and educational environments.

## 1 Introduction

Autism Spectrum Disorder (ASD) is characterized by difficulties in social communication and interactions and the presence of restricted and repetitive behavior patterns [35]. The prevalance of ASD continues to rise, affecting one in 36 children in the United States[7]. There is a broad spectrum of abilities observed within this population. Many individuals with this diagnosis live a fulfilling independent life, while others require 24-hour support to function and maintain safety. Professional and parent advocates recently began referring the latter group as having “profound” autism. This diversity underscores the importance of a personalized approach to care and intervention, tailored to the unique needs and strengths of each individual with ASD.

### 1.1 High-Risk Behaviors

In addition to diverse neurodevelopmental challenges associated with ASD, challenging behaviors often emerge that interfere with daily functioning. [22, 34, 5]. A range of impact exists from mild disruption to high-risk behaviors that have the potential to cause injury or even death such as aggression, elopement (wandering or bolting away from supervision), pica (ingestion of inedible objects or poisonous fluids), and self-injurious behaviors (SIB).

Self-injurious behaviors have the potential to result in tissue damage, broken bones, and contusions. Concussions and retinal detachment resulting in permanent loss of vision may occur in the case of head-directed SIB. Emerging research into sports where athletes sustain frequent hits to the head shows an association between repeated head trauma and long-term neurological conditions such as chronic traumatic encephalopathy (CTE) [28]. Outcomes of CTE include mood disorders, cognitive decline, memory problems, poor impulse control, and aggression [40]. It is logical that this line of research applies to head-directed SIB as well with an even more detrimental impact when there is an existing ASD disability.

Elopement is another high-risk behavior with potentially tragic outcomes. For individuals who lack safety awareness, elopement has resulted in drowning, death from hyperthermia or hypothermia, and being struck by vehicles or trains [27].Restrictive environmental modifications are often needed to ensure safety such as fencing, window locks or blocks, door alarms, and interior bolt-locks. Some people with elopement may require GPS or radio tracking devices.

Aggression may cause injury to both the person engaging in the behavior and those who are intervening to maintain safety. Injuries from aggression result in extended time away from work or permanent disability for workers in an industry already plagued by staffing shortages and frequent turnover. Workman’s compensation claims can be costly for agencies serving those with high-risk behaviors. Aggression often leads to the need for physical interventions that can be traumatizing and harm trust and relationship building with care partners.

While in some cases, the underlying causes of these high-risk behaviors may be difficult to determine, many times they stem from a combination of environmental and internal factors along with underlying skill deficits, particularly in communication and self-regulation. As part of best practices, behavioral clinicians conduct functional behavior assessments to evaluate variables that contribute to a behavior of concern. Despite this technology, it is not always possible to predict whether a particular set of circumstances will trigger a behavior on a given day or if a mild versus a more severe form of a behavior will occur. Understanding and addressing high-risk behaviors is vital, as they significantly impact the quality of life for many individuals with ASD and their care partners.

### 1.2 Medical Events

Reflecting the complexity of ASD, individuals with ASD frequently encounter a spectrum of co-morbid medical issues, including sleep disturbances [2, 3, 36, 8], sensory sensitivities [42, 6], gastrointestinal disorders [16, 9], and seizure disorders [44, 13]. Co-morbid psychiatric conditions are also common among those with ASD[31]. These co-morbid medical conditions increase complexity, adding to treatment challenges and significantly affecting the quality of life of those with ASD [25]. Among these, seizure disorders represent a particularly complex challenge, standing out for their critical implications on health and well-being compared to other co-morbidities [15, 45]. The high frequency of seizure episodes in individuals with ASD [15] necessitates urgent and effective management strategies. Immediate use of rescue medications is often essential for effective control of these episodes. Given the additional challenges individuals with ASD face, such as communication and behavioral issues, seizures introduce further complexity to their care. There’s a critical need for swift intervention during seizures to mitigate their effects. Enhancing seizure prediction could lead to efficient management, diminishing the impact of seizures on both healthcare systems and the individuals’ well-being, thus highlighting the significance of predictive models and well-structured care plans in improving the management of ASD.

Research highlights a significant correlation between the incidence of seizures and the diagnosis of ASD [20, 29], particularly among children and adolescents. A hypothesis could be that sensory sensitivities, often observed in individuals with ASD, might serve as predictive indicators for seizure episodes, suggesting a connection where heightened sensory processing challenges precede seizure activity [26]. This insight into the relationship between sensory sensitivities and seizures underscores the critical need for predictive models that can preemptively identify and mitigate these high-risk events.

Additionally, children with ASD are notably more likely to be admitted to the hospital following an emergency department visit for seizure-related disorders, with 4.7% [45]of such visits related to seizure disorders. This statistic not only reflects the severe impact of seizures on this population but also points to the broader implications for healthcare systems and families [43, 21]. The ability to predict and manage seizure episodes in individuals with ASD could significantly reduce emergency department visits and hospital admissions, thereby improving outcomes.

Various approaches have been explored to predict seizures, with a primary method involving the analysis of electroencephalography (EEG) data to identify patterns or anomalies indicative of upcoming seizures [30, 10, 39, 32, 11, 33, 24, 1, 33]. Beyond traditional EEG methods, recent advancements in seizure forecasting have leveraged machine learning to enhance algorithmic accuracy and have investigated non-EEG-based indicators, incorporating heart rate variability [17], in-ear EEG signals [18], and electromyography from biceps muscles [4], environmental factors [37], and cyclic seizure patterns [19, 14]. Additionally, stress levels, heart rate variability, and sleep quality have been identified as promising noninvasive markers for monitoring seizure susceptibility over extended periods [41].

### 1.3 High-Risk Event Prediction

Despite the promising advancements in models for predicting high-risk medical and behavioral events, several limitations and challenges hinder their application, particularly among children with ASD. Firstly, the continuous and long-term use of EEG devices for seizure detection can be impractical and not well-tolerated, especially for children with the sensory sensitivities common in ASD. These sensitivities may lead to discomfort or distress, making consistent device wear challenging. Secondly, challenging events forecasting aims to estimate the likelihood of an occurrence of the event on any given day, offering a potentially more practical approach over predicting the precise timing of the next seizure.

The necessity for non-invasive and sensory-friendly alternatives is therefore apparent, as traditional EEG devices, with their wires and electrodes, can be particularly bothersome for individuals who are sensitive to tactile sensations. This challenge underscores the need for new approaches that can accommodate the unique needs of children with ASD, ensuring that seizure prediction methods are both effective and comfortable for the population. Secondly, available techniques mainly focused on developing prediction algorithms to manage episodic seizures or high-risk behavioral events. Yet, the application of these algorithms in clinical settings has been scarcely examined [12]. In contrast, forecasting aims to estimate the likelihood of a seizure occurring on any given day, offering a potentially more practical approach than predicting the precise timing of the next seizure.

In this work, we introduce a novel model that utilizes the history of challenging behaviors from over 300 children with profound ASD over a period of nine years to assess the risk of high-risk medical and behavioral events the following day, including seizure episodes, SIB, aggression and elopement. To the best of our knowledge, this is the first work aimed at leveraging the relationships between different challenging behaviors in temporal space to form a more reliable prediction. We developed an AI-driven model using a convolutional neural network (CNN) to create a mapping between historical behaviors and this likelihood.

## 2 Data Collection

### 2.1 Participants

The study was conducted at The Center for Discovery in New York State, a facility that offers educational, medical, clinical, and residential services to individuals with severe and complex disabilities, including ASD. The participants in this study needed residential care because they were unable to succeed in less restrictive settings due to the severity and complexity of their conditions.

We analyzed an existing set of deidentified data routinely collected as part of standard operating procedure at a residential center serving children and adults with significant disabilities including profound autism. All those residing at the center have an intellectual disability, with most functioning in the moderate to profound range. The majority of the residents have limited verbal communication and need assistance to complete activities of daily living. Those placed at the center were unsuccessful in other settings due to their intensive support needs and the severity and complexity of their disability. Only de-identified data from residents previously diagnosed with ASD were included in this study.

The study utilized two datasets. The first dataset encompassed data on challenging behaviors, continuously collected by trained direct care staff across three shifts within a 24-hour period: morning (7:00–3:00), afternoon (3:00–23:00), and overnight (23:00–7:00), for 331 individuals over nine years. This dataset included a wide range of labels for behaviors observed. The most common behaviors tracked included Aggression, Disruptive Behavior, Elopement, Self-Injurious Behavior (SIB), Impulsive Behavior, Agitation and Mouthing/Pica. Examples of other behaviors labels included Property Destruction, Task-Refusal, Inappropriate Touch, and those falling into a category associated with restricted and repetitive behaviors such as Ritualistic Behaviors and Sensory/ Stimulation behaviors. The second dataset included the duration of seizure episodes during the daytime and the recovery time from after each episode which included recorded data for 177 individuals. This study was approved by both the CFD’s and Emory’s (Ethical) Institutional Review Board (STUDY00003823: ‘Predicting Adverse Behaviour in Autism’).

## 3 Methods

### 3.1 Preprocessing

In the preprocessing stage for the challenging behavior dataset, we began by identifying the top 7 most prevalent behaviors across our entire study population as indicated above. Any behaviors not fitting these categories were grouped under the label Other, resulting in a framework with 8 distinct behavior types for each recorded episode (i.e., an event that occurred in the morning, afternoon, or evening shifts). By aggregating the labels across different times of the day, we generated a binary vector with 8 entries for each day for every participant. Given our focus on predicting aggression, SIB and elopement as high-risk behavioral events, we excluded records from individuals without any incidents of agression, SIB or elopement. Consequently, our refined dataset included records from 259 individuals with Aggression, 177 with SIB and 95 with elopement. For the seizure dataset, we initially identified individuals featured in both datasets. Subsequently, we introduced a binary feature indicating the presence or absence of a seizure episode for a given individual on a specific day, resulting in a binary vector with 9 entries for each day. This process yielded a dataset that included 55 individuals.

**Figure 1:**
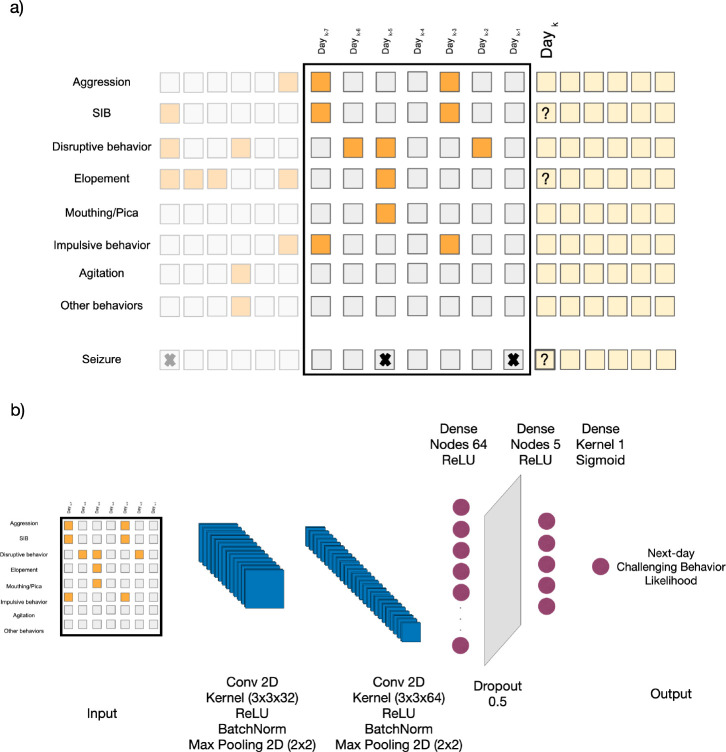
Schematic of the predictive analysis workflow. a) The feature vector is composed of seven selected behaviors, determined to be most prevalent across the study population, along with any other observed behaviors, each represented in binary form. This information forms a two-dimensional feature vector covering a timespan of the past seven/fourteen days, with the label indicating the occurrence of challenging behaviors and seizure episode on the subsequent day. Cells with lower opacity represent records from previous days, the feature vector consists of the cells enclosed by the black rectangle, and the yellow cells represent the upcoming days for which we aim to predict the presence of high-risk events. b) The diagram illustrates the deep learning model designed for the binary prediction of the occurrence of high-risk behaviorial and medical events on the following day. The model architecture includes two-dimensional convolutional layers, batch normalization, max pooling, dense layers with ReLU activation, a dropout layer for regularization, and a final dense layer with a sigmoid activation function for outputting the likelihood for an individual displaying a given behavior (e.g., self-injurious behavior, elopement, or seizure episode) the following day.

In forming the input features for each participant, as illustrated in Fig. 1(a), we used two time windows. The first approach involved using data from the 7 days leading up to an event to forecast aggression/SIB/elopement/seizure occurrences on the following day. The second approach extended this time window by using data from the previous 14 days for the same task.

### 3.2 Prediction Model

In this paper we used a CCN architecture with two-dimensional convolutional layers to extract spatial features effectively. It integrates batch normalization layers to maintain stable learning conditions and employs max pooling layers to decrease data dimensionality. The architecture is further enhanced with dense layers activated by the ReLU function, which are instrumental in identifying nonlinear relationships. To mitigate the risk of overfitting, a dropout layer is incorporated. The architecture culminates in a dense layer activated by a sigmoid function, designed for predicting the likelihood of events, as depicted in Fig. 1(b).

### 3.3 Evaluation Metrics

The training procedure for these models involved allocating 80% of the data for training, while the remaining 20% was set aside for testing purposes. This approach, including both training and evaluation, was conducted in a subject-specific manner, aiming to preserve the temporal causality while assessing the predicting scores through a individual-specific analysis.

To evaluate the effectiveness of our model, we utilized area under the receiver operating characteristic curve (AUROC), area under the precision-recall curve (AUPRC), accuracy, and *F*_1_ score. These metrics were computed through macro-averaging across all subjects and evaluations are presented as mean ± standard deviation for individuals. Basically, we computed the metrics for each individual and found the population mean and standard deviation across the population of individuals. For assessing the statistical significance of the model’s accuracy over randomness, we chose accuracy as the primary metric due to its relevance to the specific classification tasks. We employed permutation testing as detailed in Algorithm 1. This method shuffles the labels of the test set to generate distributions of accuracy under the null hypothesis that our model’s performance is comparable to random guessing (i.e., prevalence aware guessing). The performance of our model is deemed statistically significant if it surpasses the accuracy benchmarks for a specific individual, as determined by comparing the actual model’s performance against this distributions.

Moreover, to quantify the improvement offered by the model, we presented the Δ accuracy which is the average increase in accuracy compared to the expected accuracy under the null hypothesis random permutations of the data. This measure illustrates the effect size for each task, indicating the practical significance of the model’s predictive capability.

**Algorithm 1:** Statistical Test

1:	Compute the model’s accuracy a on the observed test set.
2:	Compute the distribution A for the model’s accuracy under the null hypothesis that the labels are independent of the data permuting the labels for the test set $m=10^{4}$ times and calculating accuracy each time.
3:	Determine the *p*-value p=Pr(A≥a)=|{b≥a:b∈A}|/m as the fraction of permutations m with an accuracy than is greater or equal to the observed accuracy a.
4:	if p<0.05:
	Reject $H_{0}$, concluding the model’s performance is statistically significant.

#### Feature importance

Here we explore the feature importance in our CNN model, designed for the binary prediction of high-risk behavioral and medical events the following day. We utilized Gradient-weighted Class Activation Mapping (Grad-CAM) [38] on the first convolutional layer of the CNN. Contrary to traditional applications that target deeper layers, focusing on the first layer allowed us to understand the initial feature extraction process directly related to the input data. Grad-CAM generates a coarse localization map, visually highlighting the significant regions in the input image that influence the model’s prediction. The method is formulated as follows:

1. Compute the gradient of the class score, $Y^{c}$ (here did the analysis for positive class c=1), with respect to the feature maps, $A^{k}$ (k here is the index of the filter), of the first convolutional layer to obtain $\frac{\partial Y^{c}}{\partial A^{k}}$.
2. Calculate the neuron importance weights, $\alpha_{k}^{c}$, through average pooling of these gradients, expressed as $\alpha_{k}^{c}=\frac{1}{Z}\sum_{i}\sum_{j}\frac{\partial Y^{c}}{\partial A_{ij}^{k}}$, where Z represents the total number of pixels (i.e., features) in a feature map, and i,j are the pixel indices.
3. Produce the Grad-CAM heatmap by applying a weighted combination of these activation maps and a ReLU function: $L_{\text{Grad-CAM}\;}^{c}=ReLU\left(\sum_{k}\alpha_{k}^{c}A^{k}\right)$.

## 4 Results

### 4.1 Performance Analysis

**Figure 2:**
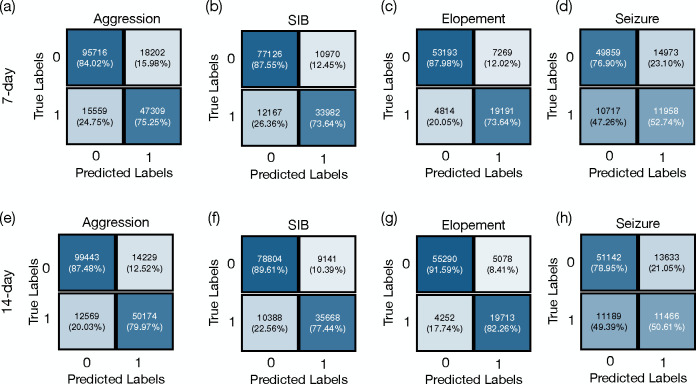
Confusion matrices for predicting aggression, self-injurious behavior (SIB), elopement, and seizure. Sub-tables (a), (b), (c), and (d) illustrate confusion matrices predict these behaviors based on a 7-day historical period. Sub-tables (e), (f),(g), and (h) predict these behaviors based on a 14-day period.

The confusion matrices presented in Figure 2 illustrate the model’s predictive performance across a cohort of individuals exhibiting high-risk behaviors. Specifically, for aggression (recorded in 259 individuals)the model demonstrated micro accuracies of 80.90% and 84.81% using historical behaviors 7-day and 14-day time windows, respectively, with corresponding micro F1 scores of 0.737 and 0.789. For SIB, observed in 177 individuals, the model achieved micro accuracies of 82.77% and 85.43%, and micro F1 scores of 0.746 and 0.785 for the same windows sizes. In predicting elopement, identified in 95 individuals, the model reached its highest micro accuracies of 85.70% and 88.94% for the 7-day and 14-day, respectively, accompanied by micro F1 scores of 0.761 and 0.809. Seizure events, reported in 55 individuals, were predicted with lower accuracies of 70.64% and 71.61%, and F1 scores of 0.482 and 0.480 for 7-day and 14-day. These results indicate that utilizing 14 days of historical data leads to slightly higher accuracy and F1 scores across all tasks.

Table 1 presents the mean and standard deviation (SD) of the macro F1 scores and macro accuracies for each behavior prediction across different time frames. For aggression, the F1 score was 0.77±0.19 over 7 days, which slightly increased to 0.80 ± 0.19 over 14 days. The model’s accuracy for predicting aggression also rose from 75.95% ± 13.94 to 78.47% ± 16.67 as the prediction window extended. In the case of SIB, the F1 scores were 0.82 ± 0.13 for the 7-day and 0.83 ± 0.18 for the 14-day predictions, with accuracies of 79.09% ± 12.94 and 80.68% ± 16.24, respectively. For elopement, there was an increase in the F1 score from 0.85 ± 0.16 at 7 days to 0.88±0.13 SD at 14 days, and accuracy improved from 81.93%±13.54 to 85.42%±11.3. The seizure predictions held steady with an F1 score of 0.77 across both periods and a slight improvement in accuracy from 69.49% ± 0.05 to 69.95% ± 0.06.

The micro-averaged scores presented in Table 1 align with the macro-averaged scores, where utilizing 14 days of historical data consistently leads to slightly higher accuracy in behavior prediction. This trend is reflected across all behaviors, with an increase in mean accuracy when the model incorporates an extended range of historical data.

### 4.2 Statistical Analysis

We employed the significance test outlined in the methods section (see subsection 3.3) to evaluate the statistical significance of the model’s performance for each individual. The number of instances where the null hypothesis (stating that the model does not predict better than permuted labels) was rejected is also noted. For aggression, significance was achieved in 233 out of 259 cases over a 7-day period and in 229 out of 257 cases over 14 days. For predicting SIB, the counts were 163 from 174 for the 7-day window and 162 from 177 for the 14-day window. With elopement, the model’s predictions were significant in 88 out of 95 cases for both 7-day and 14-day periods. Lastly, for seizure predictions, the null hypothesis was rejected in 53 out of 55 cases for the 7-day period and in 51 out of 55 cases for the 14-day period. The delta accuracy (proxy of the effect size) reinforces the conclusion that a 14-day historical data window significantly enhances the model’s accuracy for predicting high-risk behaviors.

Table 1 also presents the results for predicting severe aggression and severe SIB in a cohort consisting of 7 individuals with severe aggression and 5 with severe SIB, selecting cases where at least 10% of the target behavior was recorded as severe. The results, as detailed in the attached table, reveal a nuanced efficacy of our predictive models. For severe aggression, over a 7-day timeframe, the model’s F1 score was modest at 0.79 ± 0.13, with an accuracy of 69.5% ± 14.95, while for a 14-day period, the F1 score showed a slight increase to 0.83 ± 0.09 with accuracy marginally improved to 73.81% ± 0.13. Severe SIB predictions were similar, with F1 scores of 0.78 ± 0.09 and 0.82 ± 0.09 over 7 and 14 days, respectively. The accuracy for these predictions also saw a modest rise from 68.38% ± 10.24 to 73.97% ± 11.36 when extending the prediction window. However, the effect sizes remained low across both behaviors, with delta accuracies ranging from 2.34 ± 1.21 to 3.25 ± 1.76, indicating that the models had minimal success in predicting severe high-risk behaviors. Significance was achieved in fewer than half of the cases for both severe aggression and severe SIB.

### 4.3 Feature Importance Analysis

**Figure 3:**
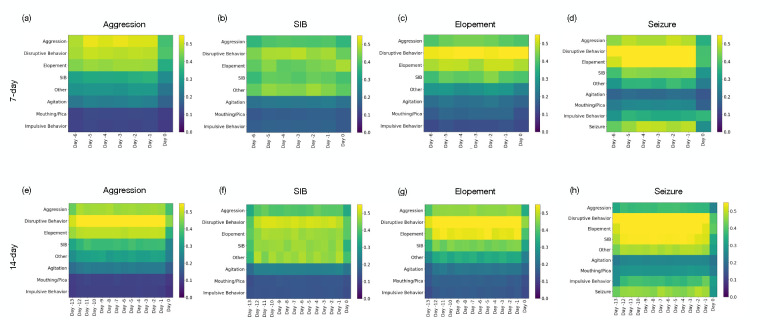
Representation of features importance using GradCAM for predicting high-risk events. Day -j refers to the *j*^*th*^ day preceding the target day for which the prediction is being made.(a), (b), (c), and (d) illustrate the feature importance rankings for predicting aggression, SIB, elopement, and seizures, respectively, based on a 7-day window. (e), (f), (g), and (h) extend the analysis to a 14-day window for the same outcomes. The comparison highlights how the predictive value of specific features shifts with the extension of the historical data period.

Figure 3 presents the impact of each feature using Grad-CAM, presented in the methods section (see 3.3); we observe that impulsive behavior plays a crucial role in predicting all high-risk events, underscoring its significance across different high-risk behaviors. Particularly noteworthy is the predictive value that the history of seizure events holds when forecasting future seizures; however, it is not the sole contributor. The analysis indicates that other behaviors, such as disruptive behavior and agitation, also exhibit a prominent predictive value in seizure forecasts. This insight is crucial as it suggests that considering a spectrum of behaviors, a multifaceted approach could enhance the accuracy of predicting seizures and other high-risk events.

The results indicates that, since such behaviors are inherently sparse, the models’ ability to predict severe high-risk behaviors is constrained. This limitation is particularly pronounced due to the lack of additional, pertinent information regarding individual subjects, such as major life events, medication usage, and comorbidities. Therefore, the practical utility of the models for predicting severe high-risk behavior without incorporating more comprehensive information or features is limited. It underscores the necessity for a more holistic approach in model development that integrates broader aspects of individual profiles to enhance predictive accuracy.

Another observation was as we extended the historical window to 14 days, illustrated in panels (e) through (h), there was a notable shift in the importance of these features. For instance, aggression remained a dominant feature for predicting SIB over both time frames, whereas impulsive behavior was prominent in the 14 days for predicting elopement. This analysis highlights how extending the historical data window can recalibrate the predictive value of specific features, which may refine our predictive models’ accuracy for severe high-risk behaviors.

## 5 Discussion

The behavioral and medical complexity of those with profound autism presents challenges for those providing care. While typical antecedents to high-risk behaviors may be identified through functional behavior assessment procedures, such antecedents may not reliably evoke the behavior of concern. Furthermore, behaviors targeted for reduction may have differing levels of intensity and corresponding impact on the person and the environment. Knowing in advance that conditions are such that a high-risk behavior or medical event is likely would allow care partners to employ additional safeguards and supports. If a seizure is predicted, a care partner may maintain constant physical contact to prevent or lessen the impact of a fall. Physical demands could be limited with allowance for more rest. When high-risk behaviors are predicted, care partners could implement preventative strategies such as environmental modifications, increased reinforcement for positive behaviors, noncontingent access to preferred people or activities, decreased demands, adapted schedules, and enhanced staffing.

**Figure 4:**
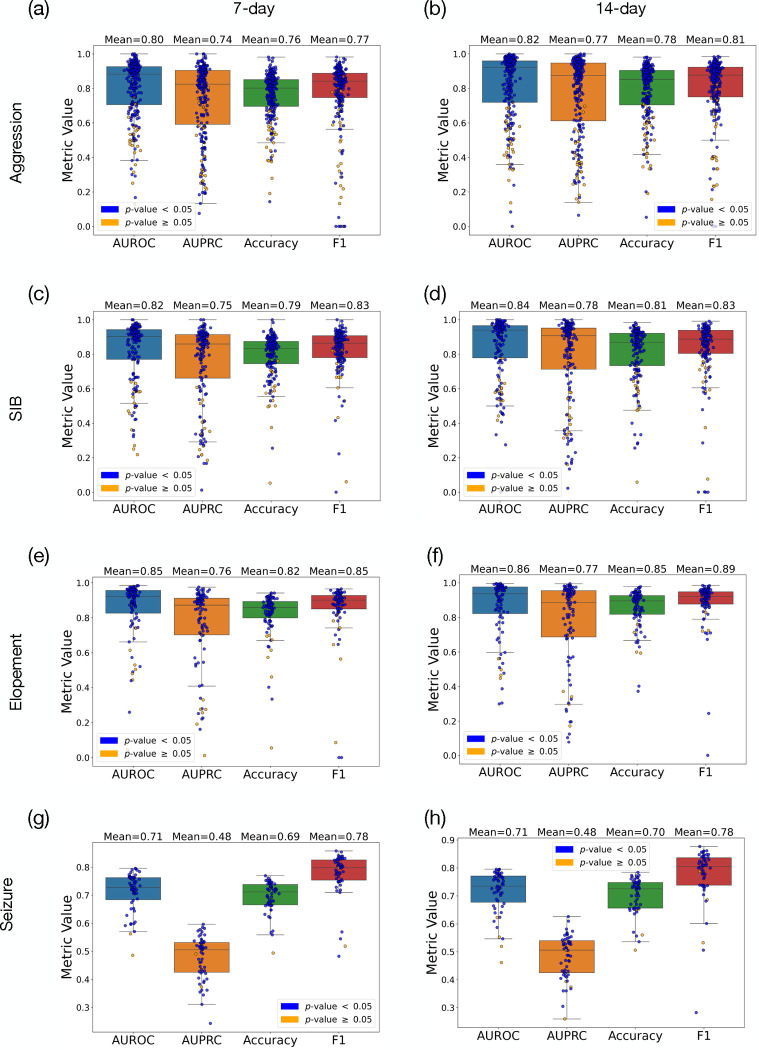
Representation of prediction results using 7-day and 14-day spans of prior historical data for predicting SIB, elopement, and seizure, presenting AUROC, AUPRC, accuracy, and F1 score. Blue and yellow circles represent cases where we achieved, and could not achieve, statistical significance, respectively. Subfigures (a), (c), (e), and (g) depict the results for aggresion, SIB, elopement, and seizure using 7-day data, while (b), (d), (f), and (h) illustrate the results for SIB, elopement, and seizure using 14-day data.

In this paper we demonstaretd a AI-driven model for predicting such high-risk behavioral and medical episodes.Table 1 and Figure 4 demonstrated a clear advantage in predictive performance when utilizing 14 days of historical data compared to 7 days. This trend was observed across all high-risk behaviors and different metrics, where both the $F_{1}$ score and accuracy show notable improvements. The extended data window likely provides a more comprehensive behavioral pattern, which enhances the model’s ability to predict subsequent high-risk behaviors with greater reliability. Although the results indicated that the null hypothesis could be rejected for over 95% of the population across different tasks, the success in predicting the severity levels of behaviors was comparatively modest. This limitation may arise from insufficient data on tasks aimed at predicting high-risk behaviors of varying severity. Since days with severe behaviors are rare, they limit the effectiveness of the predictive model in these instances.

To analyze the impact of improved prediction accuracy, we explored how enhancements in Δ Accuracy for predicting high-risk events could enhance preventive care. Specifically, the Δ Accuracy values of 10.32% for Seizure, 14.86% for Aggression, 15.78% for SIB, and 18.19% for Elopement demonstrated the model’s improved capability over the baseline. When applied in a clinical setting, these gains significantly influenced the identification of children most at risk. The enhancements provided by the improved models were quantified under a scenario with 300 individuals, aiming to identify the 30 most susceptible to high-risk events. The calculations showed that while the baseline model was expected to correctly identify 21 children, the improved models for Seizure, Aggression, SIB, and Elopement correctly identified 24, 26, 27, and 27 children respectively. This increase in correct identifications, especially for Aggression, SIB, and Elopement, significantly improved the likelihood of correctly identifying those most at risk. This precise targeting enabled more effective allocation of preventive interventions, ultimately improving outcomes for the most at risk children.

Exploring practical implementations of advancements that improve prediction accuracy for seizure episodes is essential to reduce risks in real-world scenarios. Enhanced seizure prediction, with a 10.32% increase in Δ Accuracy, marks an improvement in detection. This is crucial in settings where intervention can prevent consequences of unexpected seizure episodes. Swift action by healthcare professionals, enabled by predictive tools, can reduce trauma or complications from seizures. Integrating this technology into patient monitoring systems permits ongoing risk assessment, creating a safer environment for those prone to seizures. Moreover, data from predictive models can guide treatment plans, fostering an epilepsy management approach that adjusts to the disorder’s variability.

The findings of this study extend the understanding of seizure prediction by highlighting not only physiological predictors but also the role of changes in behaviors and moods as indicators of imminent seizure episodes. This is supported by a comprehensive online survey conducted across Canada, involving participation from 196 patients and 150 caregivers. According to the survey results, a notable percentage of both patients and caregivers, specifically 12.2% and 12.0% respectively, reported the ability to anticipate seizures. Remarkably, some were able to make these predictions up to 24 hours in advance. They identified a range of preictal symptoms, including dizziness, shifts in mood, and cognitive disturbances, as reliable signs of the approach of a seizure episode. These observations underline the potential for non-physiological cues to play a critical role in seizure forecasting, offering valuable time for preemptive measures [23].

Another observation from this study, as illustrated in Figure 3, relates to the influence of previous days and distinct behaviors on the prediction of high-risk events. It was observed that each behavior or medical event, such as seizure, played a significant role in predicting occurrences of the same type, indicating a cyclic pattern. This suggests that historical instances of a particular behavior tend to recur, highlighting the potential for targeted preventive interventions. Moreover, disruptive behavior was found to have a notable impact on the prediction of all high-risk events. This widespread influence could be attributed to the pervasive nature of disruptive behaviors, which are often symptomatic of underlying instability or distress that may precipitate a variety of high-risk events. These observations underscore the complexity of behavioral dynamics and the critical need for holistic approaches in predictive modeling and intervention strategies and CFD provides a good environment to deploy and evaluate the system.

## 6 Conclusion

This study demonstrated the feasibility of using a deep learning-based algorithm to predict high-risk behaviors and seizure episodes in children with profound ASD. By analyzing an extensive dataset covering nine years of behavior and seizure data from 331 children, our model has shown significant potential in prediction of high-risk events. The developed model attained accuracies of 78.4%, 80.68%, 85.43%, and 69.95% for predicting the next-day occurrence of aggression, SIB, elopement, and seizure episodes, respectively. The results were proven significant for more than 95% of the population for all high-risk event predictions using permutation-based statistical tests.

Our findings highlight the interplay between behavioral and medical challenges in profound autism, underscoring the critical need for advanced predictive tools. The ability to forecast high-risk behaviors and medical events could revolutionize the care paradigm, enabling personalized interventions and support that are anticipatory rather than reactive. This proactive approach allows for the optimization of environmental and staffing adjustments, the implementation of preventive measures against seizures, and a reduction in the potential impact of high-risk behaviors on individuals and their environments. Such a model could also help minimize high-risk behaviors, thereby reducing caregiver injuries that lead to prolonged absences, staffing shortages, and costly workers’ compensation claims.

## Figures and Tables

**Table 1:** Performance metrics for Predicting next day high-risk behavior using the historical records of previous 7 and 14 days (*Mean ± SD*). Significance Achieved represents the number of cases in the population for which we could reject the null hypothesis. Δ Accuracy indicates the margin by which our model outperforms the baseline.

Behavior	Days	*F*_1_ Score (*Mean ± SD*)	Accuracy (*M ± SD*)	Significance Achieved	Δ Accuracy (*M ± SD*)

Aggression	7 days	0.77 ± 0.19	75.95% ± 13.94	233 from 259	12.95 ± 10.27
Aggression	14 days	0.80 ± 0.19	78.4% ± 16.67	229 from 259	14.86 ± 11.65
SIB	7 days	0.82 ± 0.13	79.09% ± 12.94	163 from 177	14.51 ± 10.94
SIB	14 days	0.83 ± 0.18	80.68% ± 16.24	162 from 177	15.78 ± 12.55
Elopement	7 days	0.85 ± 0.16	81.93% ± 13.54	88 from 95	16.51 ± 12.29
Elopement	14 days	0.88 ± 0.13	85.42% ± 11.3	91 from 95	18.19 ± 12.89
Seizure	7 days	0.77 ± 7.15	69.49% ± 5.35	53 from 55	10.25 ± 4.93
Seizure	14 days	0.77 ± 0.10	69.95% ± 0.06	51 from 55	10.32 ± 5.29
Severe Aggression	7 days	0.79 ± 0.13	69.5% ± 14.95	3 from 7	2.34 ± 1.21
Severe Aggression	14 days	0.83 ± 0.09	73.81% ± 0.13	3 from 7	0.68 ± 1.28
Severe SIB	7 days	0.78 ± 0.09	68.38% ± 10.24	2 from 5	4.13 ± 2.21
Severe SIB	14 days	0.82 ± 0.09	73.97% ± 11.36	3 from 5	3.25 ± 1.76
